# Artificial intelligence in the diagnostic imaging of developmental dysplasia of the hip: a systematic review

**DOI:** 10.1530/EOR-2024-0165

**Published:** 2026-04-07

**Authors:** Abith Ganesh Kamath, Saran Singh Gill, Hussayn Shinwari, Kapil Sugand

**Affiliations:** ^1^ Faculty of Medicine, Imperial College London, London, United Kingdom; ^2^ Faculty of Medicine, St George’s University of London, London, United Kingdom

**Keywords:** artificial intelligence, developmental dysplasia of hip, congenital dysplasia of hip, imaging, radiography, ultrasonography, accuracy, sensitivity, specificity, predictive value

## Abstract

**Purpose:**

- With the increased challenges in diagnosing DDH using traditional ultrasound imaging methods, accurate diagnosis is essential. This study assesses the effectiveness of AI in the imaging-based diagnosis of DDH through a systematic review.

**Methods:**

- This review was conducted in accordance with PRISMA (Preferred Reporting Items for Systematic Reviews and Meta-Analyses) guidelines and registered in Prospero (registration ID: CRD42024563606). A comprehensive search was conducted across Ovid MEDLINE, PubMed, Embase, the Cochrane Central Register of Controlled Trials, and the Cochrane Database of Systematic Reviews. Studies were screened using selection criteria, and quality was assessed using standardised tools. Thematic content analysis was also performed. Of the 32 studies identified, 19 were included, with 15 undergoing quantitative analysis. The main outcome measures were sensitivity, specificity, accuracy, AUROC, PPV, and NPV. Median, median absolute deviation, Bonett–Price 95% confidence intervals, maximum, minimum, and interquartile ranges were calculated and presented in a box-and-whisker diagram.

**Results:**

- In the 19 included studies, the median sensitivity was 90.0% and specificity was 93.2% across 36,907 patients. Fifteen studies reported diagnostic accuracy, with a median of 92.6%. Accuracy rates ranged from 79.2 to 99%. The most common model architecture was mask R-CNN. Four studies (21%) were judged to have a high risk of bias using the QUADAS-2 tool.

**Conclusions:**

- AI technologies hold significant potential for enhancing the diagnostic accuracy of DDH. However, existing variability and bias across studies highlight the need for further standardisation and validation.

## Introduction

Developmental (or congenital) dysplasia of the hip (DDH/CDH) is a common disorder in infants with an incidence of approximately 10 per 1,000 live births in the UK and USA (1). This condition encompasses several abnormalities, including instability, acetabular dysplasia, subluxation, and dislocation (2). Evidence suggests that DDH is the main cause of hip arthroplasty in young people (3).

The gold standard for diagnosing DDH through imaging is with ultrasound (4). Evidence indicates that Graf’s method is the most effective (5). Errors in ultrasound interpretation can be influenced by various factors, including inexperienced clinicians, fatigue, interruptions, suboptimal viewing conditions, and time constraints (6). Sonographers must manually identify a series of anatomical markers to estimate Graf’s α and β angles, femoral head coverage, and pubofemoral distance, making this process challenging (7). Subsequently, there have been misdiagnoses reported in half of the infant screenings and three quarters of the neonatal screenings (8).

The application of artificial intelligence (AI) in the healthcare industry is exponentially increasing, enhancing diagnostic processes and prognostication of clinical outcomes (9). This has been demonstrated in breast cancer diagnoses, with algorithms reaching pathologist-level accuracy (10). AI has the capability to streamline healthcare to provide patients with more accurate, efficient, and personalised diagnostic imaging (11). AI can also be used as a tool for triage and screening and reducing intra-observer variability between radiologists (12).

AI can be trained to identify key landmarks in hip scans and therefore calculate Graf’s α and β angles to assign type of severity, reducing errors and improving efficiency (13). A 2019 Canadian study found a 26% workload increase over 12 years, suggesting that AI could help address this, especially given the rise in imaging volume over the same period (14). The key advantage of AI is that it solely depends on data rather than domain expertise, which tend to take years to develop (15).

### Aim

To compile a systematic review observing studies looking at the performance metrics of diagnosing DDH/CDH using imaging modalities.

## Methodology

### Search strategy

This systematic review was conducted in accordance with the Preferred Reporting Items for Systematic Reviews and Meta-Analyses (PRISMA) guidelines (https://www.bmj.com/content/339/bmj.b2535) and registered in Prospero (registration ID: CRD42024563606). A comprehensive literature search was performed across Ovid MEDLINE, PubMed, Embase, and the Cochrane Central Register of Controlled Trials, including the Cochrane Database of Systematic Reviews, on 27 May 2024. The search included studies published from 1980 onwards to ensure comprehensive coverage of both early and recent literature on AI applications in developmental dysplasia of the hip (DDH).

Medical Subject Headings (MeSH) and relevant free-text terms were combined using Boolean operators. The primary search terms included: “Developmental Dysplasia of the Hip”, “Congenital Dysplasia of the Hip”, “Artificial Intelligence”, “Machine Learning”, and “Deep Learning”. References of all included studies were also screened manually using a snowballing technique to identify any additional eligible publications.

### Selection criteria

A broad search strategy was adopted to capture the full scope of emerging evidence on AI-assisted DDH diagnosis. Two reviewers independently screened titles and abstracts, followed by full-text evaluation to determine eligibility. Studies were included if they were published in English, involved paediatric human populations, were available in full text, and evaluated developmental or congenital dysplasia of the hip. Studies were excluded if they were non-English, non-peer-reviewed, or categorised as grey literature. Animal studies, research on non-dysplastic hip pathology, and publications limited to conference proceedings, abstracts, or books were also excluded.

Study selection was guided by the PICO framework. The population comprised paediatric patients. The intervention involved the incorporation of AI or machine learning models for diagnosing DDH using imaging modalities. The comparison was the diagnostic performance of conventional imaging interpretation without AI assistance, considered the clinical gold standard. The outcomes included the number of patients analysed, imaging modality used, and key diagnostic performance measures: sensitivity, specificity, accuracy, area under the receiver operating characteristic curve (AUROC), positive predictive value (PPV), negative predictive value (NPV), F1 score, and precision.

### Data extraction and analysis

Data were extracted independently by two reviewers and verified for accuracy. Extracted variables included study design, sample size, AI model architecture, imaging modality, and diagnostic performance metrics.

Given the heterogeneity of study designs and outcome reporting, a qualitative synthesis was performed, supported by descriptive statistical analysis. For quantitative outcomes, median values, interquartile ranges (IQRs), and 95% Bonett–Price confidence intervals were calculated, with results rounded to one decimal place. When multiple values for a diagnostic metric were reported within a study, pooled estimates were used for the final analysis. A box-and-whisker plot was generated to visualise the distribution of diagnostic performance metrics across studies.

### Quality assessment

**Figure 1:**
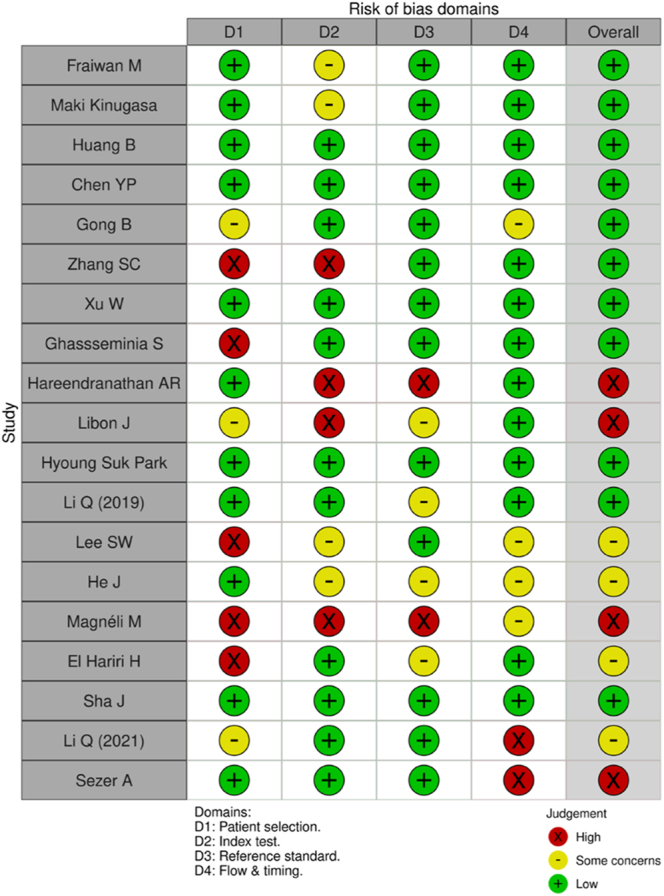
ROBVIS analysis using QUADAS-2. Data in this figure are drawn from the 19 included studies in this review (Ref. 8, 13, 16, 17, 18, 19, 20, 21, 22, 23, 24, 25, 26, 27, 28, 29, 30, 31, 32).

The methodological quality and risk of bias of included studies were assessed using the Quality Assessment of Diagnostic Accuracy Studies (QUADAS-2) tool (17). Both reviewers conducted the assessments independently, resolving discrepancies through discussion and consensus. The results were visualised using the ROBVIS tool (18) (Fig. 1). Studies reporting only qualitative data were excluded from quantitative bias assessment due to incompatibility with the QUADAS-2 framework.

## Results

### Performance breakdown of developmental dysplasia of the hip models

Table 1 summarises the performance of 19 AI models based on the categories of sensitivity, specificity, accuracy, AUROC, PPV, NPV, F1 score, and precision. Sensitivity was the most popular metric being included in 15 out of the 19 studies, ranging from 35.3 to 98%. Of the studies that include sensitivity, only Magnéli *et al.* (32) failed to measure specificity. Accuracy was also a popular metric in 10 out of the 19 studies, with reported rates up to 99.4%. AUROC, despite being measured in only 5 of the studies, remained very high with all studies above 90%. PPV, NPV, precision, and F1 score were rarely included overall, with only one study utilising the F1 score and two using precision. In terms of diagnostic performance, three models (Zhang *et al.* (29), Sha *et al.* (33), Huang *et al.* (23)) are at the forefront with outstanding sensitivity, specificity, accuracy, and AUROC.

**Table 1:** Table of results. Data are presented as percentages. Data in this table are drawn from the 19 included studies in this review (8, 13, 16, 17, 18, 19, 20, 21, 22, 23, 24, 25, 26, 27, 28, 29, 30, 31, 32).

Study	Year	Pts, *n*	Ix	Sens	Spec	Acc	AUROC	PPV	NPV	F1	Prec	Country	AI model
Fraiwan *et al.* (24)	2022	354	X	100	94.3	96.3	93.57			95	90.6	Jordan	DarkNet53
Kinugasa *et al.* (26)	2023	191	U	98	98.1			84.5	99.8				SqueezeNet, MobileNet_v2, EfficientNet
Huang *et al.* (23)	2023	209	U	90.56	100	98.64		100	98.44			China	DDH.Net
Chen *et al.* (13)	2024	921	U									Taiwan	U-Net (modified)
*α* < 60°				88.2	80.3	84.8	93.7	85.4	83.9				
*α* < 50°				97.5	62.5	94.9	97.4	97.0	66.7				
*β* > 77°				35.3	99.8	97.2	90.4	85.7	97.4				
Gong *et al.* (22)	2022	758	U	86.54	85.23	85.89						China	TML-derm
Zhang *et al.* (29)	2020	1,138	X	95.50	99.5	99	97.5					China	ResNet-101
Xu *et al.* (28)	2022	1,265	X									China	Mask R-CNN + HRNet
SL: Lt				91.7	92	91.1							
SL: Rt				94.7	96.3	87.5							
LEA: Lt				88.7	86.8	92.9							
LEA: Rt				89.5	88.7	92.6							
AS: Lt				86.5	84.3	90							
AS: Rt				85.7	85	87.9							
Ghasseminia *et al.* (20)	2022	240	U	83.1–100	16–21							Canada	MEDO-Hip
Hareendranathan *et al.* (19)	2022	107	U			89							MEDO-Hip
Libon *et al.* (21)	2023	306	U									Canada	MEDO-Hip
Park *et al.* (25)	2021	2,601	X	98	98.1			84.5	99.8			South Korea	
Li *et al.* (27)	2019	11,574	X	83.3	81.9	79.2						China	Mask R-CNN + ResNet
Lee *et al.* (8)	2021	921	U									South Korea	Mask R-CNN
He *et al.* (31)	2022	216	U	87.8	89.3		93.8					China	
Magnéli *et al.* (32)	2024	571	X	81.1							96.8	Multiple*	ImageNet
Model 1						92.2							
Model 2						83.3							
El Hariri	2021	34	U									Canada	3D U-Net + 3D ResNet
Sha *et al.* (33)	2023	3,247	X	98.6	100	99.4	99.3					China	Mask R-CNN ResNet
Li *et al.* (18)	2021	13,228	X									China	Mask R-CNN
Sezer & Sezer (34)	2020	675	U	96.17	98.02								GoogleNet and AlexNet

AS, acetabular source; LEA, lateral edge of acetabular; Lt, left; Rt, right; ICC, intraclass correlation coefficient; Sens, sensitivity; Spec, specificity; Acc, accuracy; AUROC, area under the ROC curve; PPV, positive predictive value; NPV, negative predictive value; Prec, precision; X, plain radiographs; and U, ultrasonography.

* Multiple countries, including Denmark, USA, Netherlands, Sweden, Japan, UK, Spain, and Norway.

**Figure 2:**
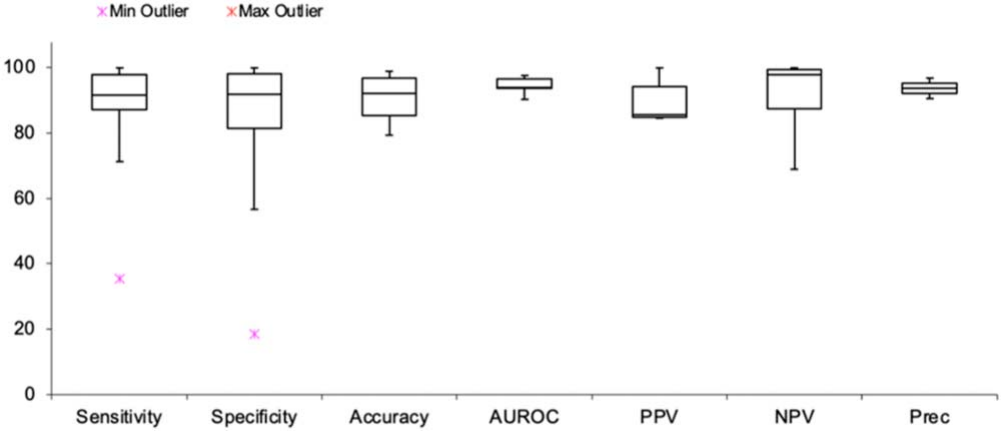
Box-and-whisker diagram (both F1 score and precision were excluded due to singular results).

A statistical analysis of the data was carried out in Table 2 and Fig. 2. Median values were highest for NPV at 97.9% and lowest for PPV at 85.6%. However, all metrics reported high median values of above 90% except for NPV. MAD was the largest in sensitivity, specificity, and accuracy as expected, due to a larger proportion of included models using these as performance evaluation metrics. Two key outliers were noted in sensitivity from Chen *et al.* (13) measuring the beta angle above 77° and specificity in Ghasseminia *et al.* (20).

**Table 2:** Statistical analysis. Data are presented as percentages.

	Pts*, *n*	Sens	Spec	Acc	AUROC	PPV	NPV	F1	Prec
Q0	34	35.3	18.5	79.2	90.4	84.5	66.7		90.6
Q1	232	87.2	81.5	85.3	93.6	84.7	87.3		92.2
Q2	746	91.5	91.8	92.2	93.8	85.6	97.9	95.0	93.7
Q3	1,599	97.8	98.1	96.8	96.5	94.2	99.5		95.3
Q4	13,228	100	100	99.0	97.5	100	99.8		96.8
95% CI									
Min	310	86.2	83.4	85.6	90.5	78.5	82.9		
Max	1,182	96.8	100	98.8	97.0	92.6	100		
MAD	528	6.0	7.1	6.3	1.8	1.1	1.9	0	3.1
Studies, *n*	16	18	19	15	7	6	6	1	2

Q0, minimum; Q1, 25% percentile; Q2, median; Q3, 75% percentile; Q4, maximum; Pts, patients; Sens, sensitivity; Spec, specificity; Acc, accuracy; AUROC, area under ROC; PPV, positive predictive value; NPV, negative predictive value; Prec, precision; and MAD, median absolute deviation.

* Total *n* = 36,907.

## Discussion

### Principle findings

**Figure 3:**
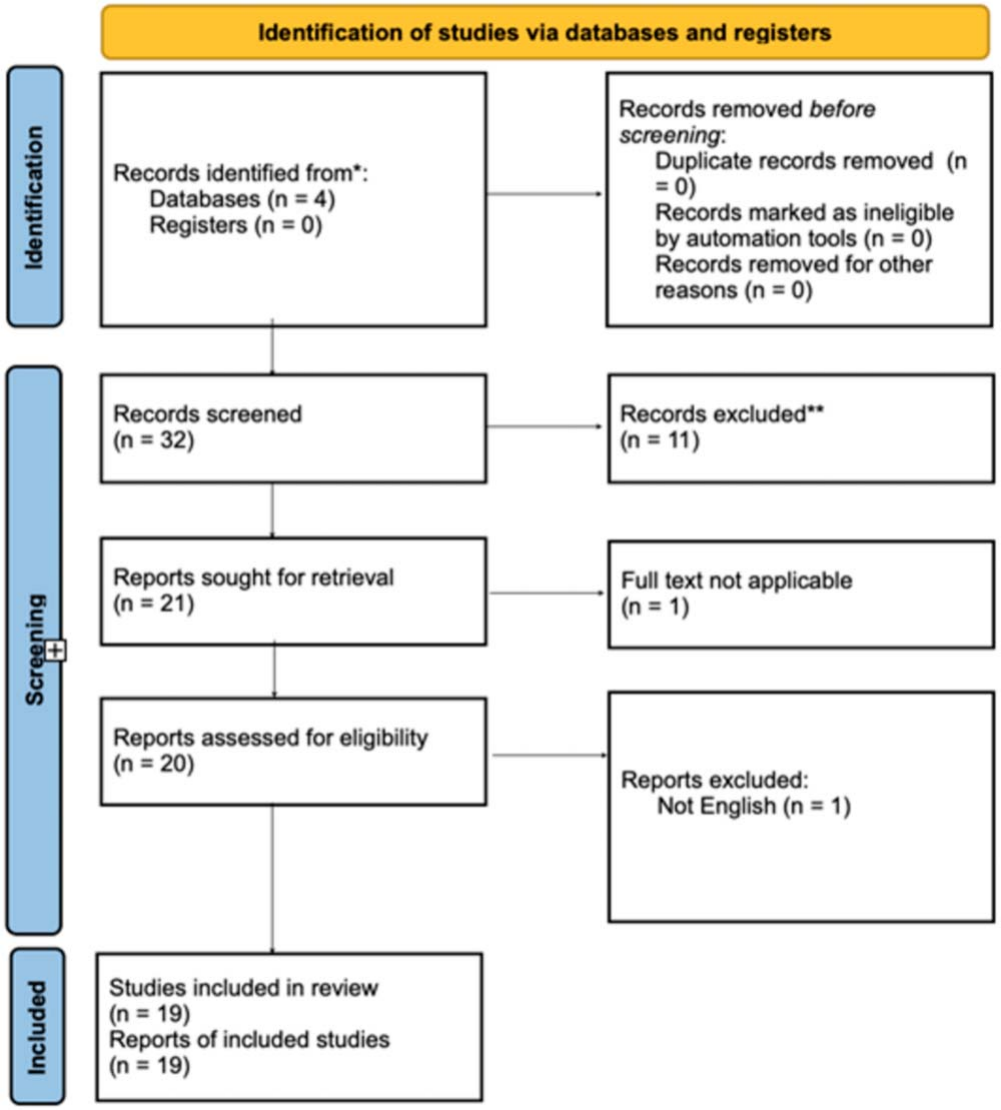
PRISMA diagram.

Nineteen studies were included in the final analysis shown in Fig. 3. Eight studies used plain radiography, and 11 depended on ultrasonography to assess a total of 36,907 patients for confirming DDH diagnosis. Main metrics consisted of accuracy, sensitivity, specificity, AUROC, positive predictive value, and negative predictive value to ascertain grades of DDH (subluxable, dislocatable, and dislocated alongside Graf angles). Only one study reported both the F1 score and precision. Four studies (21%) were assessed as having a high risk of bias, and another four (21%) were rated as having some concerns, while the majority (58%) were judged to have a low risk of bias. Only one outlier was found, that too within sensitivity. Relative to all metrics, PPV had the lowest median with the largest 95% confidence interval but smallest MAD.

### Thematic content analysis

#### Theme 1: deep learning and AI

All included studies emphasised the use of deep learning and AI to enhance the diagnostic accuracy of DDH. They leverage convolutional neural networks (CNNs) and other deep learning models to analyse ultrasound and plain radiography to identify DDH with high accuracy. The studies highlighted the potential of AI to match or exceed the performance of human experts in diagnosing DDH. Models typically used the mask R-CNN framework to detect key anatomical landmarks through object identification and image segmentation.

Of the 19 studies, 12 studies measured this using the performance evaluation metrics of sensitivity and specificity with some also outlining accuracy, AUROC, PPV, and NPV. Other studies had more unorthodox evaluation metrics with Lee *et al.* (8) using intraclass correlation coefficient (ICC) and Li *et al.* (18) comparing the time taken for the AI model to measure the lateral centre-edge (CE) angle compared to subspecialists in the field. The ICC is a descriptive statistic that tells us how similar the observations within groups are to each other, with Lee *et al.* (8) outlining the inter-rater agreement between doctors and AI models at calculating the Graf’s alpha and beta angles required to diagnose DDH. AUROC is a significant measure and is argued to be more effective than accuracy as it measures the effectiveness of the AI models by calibrating the trade-off between specificity and sensitivity, thus making it robust to class imbalance.

Hareendranathan *et al.* (19) developed a 10-point quality score for evaluating AI models, offering valuable insights into how image quality impacts the AI assessment of the MEDO-Hip model. This study revealed that the algorithm performed worse with lower-quality images, a challenge less likely to occur with expert radiologist evaluation (17). While these novel metrics are beneficial, they introduce significant heterogeneity between studies, complicating direct comparisons.

#### Theme 2: training and validation

Each study involves training deep learning models on a large dataset of medical images and validating the models’ performance on separate test datasets. Transfer learning was utilised involving fine-tuning pre-trained networks to improve diagnostic accuracy with less computational effort compared to training models from scratch. Among the included studies, the MEDO-Hip model (Hareendranathan *et al.* (19), Ghasseminia *et al.* (20), Libon *et al.* (21)) (an app interpreting cine-sweep images obtained from a handheld Philips Lumify probe) was used the most, which employs a U-Net-like CNN model to identify key structures in the femoral head and acetabulum that are important for DDH diagnosis alongside key elements of Graf’s methodology.

In the study conducted by Gong *et al.* (22), the TML-DERM model was used, which consisted of a B-mode ultrasound (BUS)-based computer-aided diagnosis (CAD) and instead contained a first-stage deep neural network (DNN) to alleviate the overfitting issue followed by a second-stage meta-learning step designed to learn the optimal combination of weak classifiers, which was initially expected to be superior to other models. Despite superior experimental results, when compared to DDH.Net (Huang *et al.* (23)) and DarkNet53 (Fraiwan *et al.* (24)), the overall metrics of sensitivity, specificity, and accuracy were lower. The main obstacle in the imaging field is obtaining high-quality training datasets of adequate size, thus reducing the risk of overfitting, generalisation, and extrapolation.

Overfitting refers to AI models performing well on training data but poorly on unseen data, including differing patient demographics, ethnicity, and phenotypes. Park *et al.* (25) reduced the risk of overfitting by augmenting the training datasets using operators such as rotation, translation, flipping, and scaling, but despite this, 10 out of the 513 cases in the training dataset were misdiagnosed by the training data algorithm. The outcome confirmed the importance of continuing to develop the architecture of neural networks, to account for misdiagnosis, and reach the proficiency of an expert radiologist.

#### Theme 3: key anatomical structures and measurements

The studies concentrate on identifying key anatomical structures of the hip, including the femoral head, acetabulum, and iliac wing, and measuring critical angles (alpha and beta) essential for diagnosing DDH using Graf’s method. Accurate identification and measurement of these structures are emphasised, as they directly impact the classification and diagnosis of DDH. However, in Kinugasa *et al.* (26), this is ambiguous as Graf 2a and 2b were deemed ‘normal’, when this was inaccurate. According to the original criteria established by Graf *et al.* (5), patients with a Graf score of 2a should be monitored, while those with a score of 2b require treatment with a Pavlik harness. In addition, Chen *et al.* (13) highlighted that the predicted beta angles had larger errors compared to alpha angles perhaps due to the difficulty in identifying the anatomical labrum, but this is a more significant factor in identifying the severity of pre-existing DDH rather than diagnosing it. This is because the beta angle has more of an auxiliary role, to determine the severity of dysplasia, with no effect on DDH diagnosis.

Aside from Graf’s method, some studies involved more novel approaches. Li *et al.* (27) instead focused on calculating Sharp’s angle, which is defined as the angle between the line connecting the lower edge of the pelvic teardrop to the upper edge of the acetabulum and the horizontal plane. The study indicated that an angle of 47° or above would suggest hip dysplasia. Despite the model having a significant accuracy compared to the three orthopaedic surgeons in the study, its overall sensitivity and specificity were far lower than those of the models using Graf’s method as their foundation, as well as there being discrepancies in the models performance between left and right hips. However, this may be due to DDH occurring on the left hip in 64% of cases, owing to increased perceived accuracy of the model (2).

Furthermore, Xu *et al.* (28) sought to measure performance based on the Tonnis and International Hip Dysplasia Institute (IHDI) classifications, indicating that the AI model showed no statistically significant difference in performance compared to intermediate surgeons. Despite the algorithm achieving this, Tonnis and IHDI classifications are designed to determine the severity of DDH rather than diagnosing the condition itself, thereby reducing the validity of the comparison. Lee *et al.* (8) and Kinugasa *et al.* (26) outlined that they studied a small number of patient samples and orthopaedic surgeons. Further research with larger, more diverse samples and additional validation studies are essential to accurately assess the efficacy and reliability of the AI model in clinical practice. Given the substantial number of studies emphasising severity over basic diagnosis, it is recommended that future research initially focus on a binary diagnosis of the condition before delving into more detailed analysis.

#### Theme 4: accuracy and reliability

The included studies demonstrated that their AI models exhibited high clinical and statistical significance for accuracy, sensitivity, and specificity in diagnosing DDH. Few studies also included the Bland–Altman test to determine agreements between machine learning (ML) models and senior experts (Huang *et al.* (23)). They compared the AI models’ performance to that of human experts (i.e. attendings or consultants, mostly orthopaedic surgeons), often revealing that AI can achieve comparable or superior results. Based on the current synthesis of data, the AI models demonstrated the capacity for excellent detection of DDH with all sensitivity and specificity metrics above 80%, notably Huang *et al.* (23) observing a specificity of 100%.

However, Kinugasa *et al.* (26) excluded poor-quality images without a standard plane before training the algorithm, thereby restricting the model’s ability to perform in conditions where there is no straight iliac line, femoral head with maximum diameter, and triradiate cartilage (35). Despite the efficiency of the AI models, the included studies confirmed the importance of uniformity when diagnosing DDH as well as its severity by plain radiography or ultrasonography. Chen *et al.* (13) demonstrated that the AI model indicated superior performance when diagnosing severe dysplasia when compared to mild to moderate dysplasia, particularly for alpha angles between 50 and 60°, thereby skewing the results.

Zhang *et al.* (29) had 7 patients misdiagnosed as DDH by the DL algorithm because of non-standard radiographs with pelvic deflection or femoral adduction; hence, it is recommended that clinicians still provide a secondary diagnosis. Moreover, Park *et al.* (25) performed a McNemar’s test on the results between the deep learning model and radiologists, and there was no significant difference in the diagnosis of DDH. However, the DL model exhibited a significantly higher AUROC compared to the less experienced radiologist.

AUROC is a graph plotted of sensitivity against 1 − specificity, which refers to the ability of the AI model to distinguish the separability between two classes, in this case, between DDH and non-DDH diagnosis. This indicates that while AI cannot yet supplant expert radiologists, it possesses a substantial value in the education of trainees. Therefore, to reduce errors, training may be performed on a larger number of outlier examples and varied datasets.

### Limitations

There is a shared recognition of the limitations associated with traditional diagnostic methods, such as labour-intensive processes and susceptibility to human error, which the AI models aim to overcome. In addition, studies such as Hareendranathan *et al.* (19) address the interobserver variability among human experts, highlighting that AI models can offer more consistent and reliable measurements. The study found that the interobserver variability in humans was mainly due to the reader’s experience with DDH ultrasonography in relation to professional speciality, highlighting that an AI model can be a useful adjunct. The AI model performed intermediate between expert and non-expert readers, thus reinforcing its use as a training tool. However, levels of accuracy were never perfect, with AI models (e.g. DDH.Net) misdiagnosing 5 Graf 2a cases as Graf 1. Therefore, it is advised that these systems should be used as adjuncts rather than replacements for expert radiologist evaluation. This recommendation primarily applies to clinical settings where diagnostic precision directly influences management decisions. As AI models continue to expand their validation cohorts, there may be the potential to operate autonomously or pre-screen in order to reduce radiologist workload. Nonetheless, at present, expert oversight remains essential to ensure diagnostic safety. Some of the studies (El-Hariri *et al.* (30), Hareendranathan *et al.* (19), Kinugasa *et al.* (26), Lee *et al.* (8)) had a small sample size (less than 5) of radiology experts measuring the Graf angle in the control group, thereby impacting external validity.

The variability among the AI models analysed in our study emphasised the need for a unified framework to evaluate their performance, which according to our systematic review remained heterogeneous in 4 of the 19 studies. Despite conducting an exhaustive search of the literature on various databases, our search results only produced 32 relevant articles, reducing the power of our study. As mentioned earlier, only 9 out of the 19 studies calculated sensitivity, specificity, and accuracy, leaving the rest difficult to include in our quantitative analysis. Moreover, every study used a different AI model with its own specific parameters and methodologies, where Fraiwan *et al.* (21) calculated the F1 score (harmonic mean of sensitivity and specificity) and precision. Precision refers to the proportion of positive predictions of DDH the AI model produces that are correct. It is also important to note that 8 of the studies analysed scans exclusively from China, which leads to the overall performance of the models becoming biased towards the characteristics of this population, as well as reducing generalisability to populations with different demographics, environmental conditions, and genetic exposures. We aimed to mitigate the inherent weaknesses of the current literature through a robust analytical strategy, although this demands cautious interpretation of the variability in some of the results.

### Future studies

Further studies include expanding datasets and improving model accuracy with incremental learning, as outlined by Fraiwan *et al.* (24). Expanding datasets is beneficial as it increases diversity of the AI model, allowing it to perform in varying patient demographics and clinical scenarios, as well as preventing overfitting. Some also emphasised the potential of AI not only to assist in diagnosis but also to provide educational tools for speciality trainees. AI can detect subtle signs of DDH, which may be pre-clinical and overlooked by less experienced clinicians (Ghasseminia *et al.* (20)). Hence, through interactive and real-time feedback, AI and ML can improve diagnostic skills to provide improved patient quality of care.

Several studies discuss the potential for integrating AI models into existing clinical systems, such as PACS, to not only provide real-time assistance but to also improve diagnostic workflows (Chen *et al.* (13)). By doing so, healthcare providers can improve speed of diagnosis and other clinical assessments, which could be a significant leap in delivering quality care. Furthermore, it is important to detect DDH early, allowing for successful intervention and management. Delayed diagnosis can result in less effective and more complex treatments (36), so implementation of AI can allow for early detection of DDH and more prompt therapeutic interventions.

## Conclusion

This is the first systematic review confirming that AI and ML models demonstrated superior performance for identifying DDH, with most of the included studies observing either comparable or more accurate results to radiology experts. However, due to limited datasets and performance variabilities, there may be clearer indications for its role as a supplementary tool for radiologists to improve workflow efficiency and training the next generation of expert diagnosticians. The findings should be interpreted with caution considering the proportion of articles presenting a low risk of bias, with the remaining presenting potential sampling and selection bias. This study recognises the key role AI can play in increasing efficiency and accuracy of DDH diagnosis in a clinical setting, reinforcing the need for a multicentre study to evaluate these models on a larger scale, involving greater patient numbers, and to also assess their cost-effectiveness and practicality. While challenges remain, the evidence suggests that AI and ML can be a useful tool for medical professionals in conjunction with pre-existing medical knowledge either to train or to aid future diagnosis. Collaboration between developers and clinicians is imperative for AI-assisted diagnosis of DDH to reach its full potential.

## ICMJE Statement of Interest

The authors declare that there is no conflict of interest that could be perceived as prejudicing the impartiality of the work reported.

## Funding Statement

This work did not receive any specific grant from any funding agency in the public, commercial, or not-for-profit sector.
